# Evaluation of the Ogawa-Kudoh method for tuberculosis isolation in two health units in Mozambique

**DOI:** 10.4102/ajlm.v9i1.929

**Published:** 2020-07-20

**Authors:** Carla M. Madeira, Khalide I. Azam, Daisy N. Sato, Celso Khosa, Nilesh Bhatt, Sofia O. Viegas

**Affiliations:** 1 National Tuberculosis Reference Laboratory, Instituto Nacional de Saúde, Marracuene, Mozambique; 2 American Society for Microbiology, São Paulo, Brazil; 3 Centro de Investigação e Treino em Saúde da Polana Caniço, Instituto Nacional de Saúde, Marracuene, Mozambique; 4 Department of the Laboratory Network and Reference Services, Instituto Nacional de Saúde, Marracuene, Mozambique

**Keywords:** Ogawa-Kudoh, mycobacterial culture, tuberculosis diagnostics, Mozambique, laboratory

## Abstract

**Background:**

Mozambique is among the highest tuberculosis, tuberculosis–HIV and multidrug-resistant-tuberculosis burden countries. Although molecular technologies are available in-country, mycobacterial isolation through culture remains an important tool for tuberculosis diagnostics and drug susceptibility testing.

**Objective:**

We evaluated the use of the Ogawa-Kudoh (OK) mycobacterial culture, a simple technique, to isolate *Mycobacterium tuberculosis* in two health units, in Maputo City, Mozambique.

**Methods:**

From May to December 2014, 122 patient samples were collected in Chamanculo General Hospital and Polana Caniço General Hospital. The specimens were first tested in the health units using the OK method and afterwards shipped to the National Tuberculosis Reference Laboratory for mycobacterial culture using the NALC-NaOH-Citrate (NALC) decontamination method followed by inoculation in Lowenstein Jensen (LJ) solid media as the reference standard.

**Results:**

Among 107 samples with valid results, 98 (91.6%) had concordant results in both methods; 9 (8.4%) had discordant results. The contamination rate was 4.1% (5/122) for the OK and 9.0% (11/122) for the NALC/LJ methods. The sensitivity of OK was 80% (95% confident interval [CI]: 51.4–94.7) and the specificity was 94% (95% CI: 85.8–97.3). The degree of agreement between both methods was moderate (Kappa: 0.68; 95% CI: 0.48–0.89).

**Conclusion:**

The OK method showed satisfactory sensitivity and specificity. The method also had a lower contamination rate when compared to the NALC/LJ. Similar to other studies in resource-limited settings, our findings showed that the OK method can effectively be implemented in settings with limited laboratory capacity to isolate tuberculosis bacteria by culture for further testing.

## Introduction

Mozambique is amongst the highest tuberculosis, tuberculosis–HIV and multidrug-resistant-tuberculosis burden countries.^1^ At present, the country has a tuberculosis laboratory network comprised of three tuberculosis reference laboratories, 85 sites performing Xpert® MTB/RIF (Cepheid, Sunnyvale, California, United States) and 400 smear microscopy laboratories. Mycobacterial culture is only performed in the three tuberculosis reference laboratories, using both solid Lowenstein Jensen (LJ) media and liquid mycobacterial growth indicator tubes for simultaneous analysis. The national testing algorithm recommends mycobacterial culture for Xpert® MTB/RIF-resistant cases and for treatment monitoring of resistant cases in order to isolate *Mycobacterium tuberculosis* for further drug susceptibility testing. Mycobacterial culture is considered the reference standard for tuberculosis diagnosis. However, one of its major constraints is sample transportation from the collection site to the reference laboratory. In most cases, long transportation distances are associated with the need for a cold chain, prompting high contamination rates in the presence of fast-growing bacteria, and that can lead to under-diagnosis of tuberculosis.

Another constraint on the performance of mycobacterial cultures is the need for high-level containment laboratories, because of testing procedures, and the need for qualified laboratory staff.^2,3,4^ These requirements come with high costs, which contribute to challenges associated with implementing mycobacterial culture in rural areas.^4^

In order to detect and treat all tuberculosis cases, it is necessary to expand simple and accessible diagnostic laboratory services, especially in resource-limited countries such as Mozambique. In 1974, Kudoh and Kudoh described a simple, rapid and inexpensive mycobacterial culture method, the Ogawa- Kudoh (OK) method,^5^ which does not require high-level laboratory containment or extensive technical training.^5^ Evaluating the applicability of the OK mycobacterial method opens up the possibility for less specialised laboratories to carry out tuberculosis culture, increasing the diagnosis capacity in the country. The aim of this study was to evaluate the accuracy and applicability of the OK method, as compared to LJ mycobacterial culture as the reference standard, for *M. tuberculosis* isolation in two peri-urban health units in Maputo City, Mozambique.

## Methods

### Ethical considerations

Ethical approval to conduct the study was obtained from the National Bioethics Committee (CNBS), Ministry of Health, Mozambique, with the reference number 368/CNBS/13. The patients were included after understanding the study procedures and signing a written informed consent form.

### Study setting

The study was conducted in two health units in Maputo City, Chamanculo General Hospital and Polana Caniço General Hospital. Both are level II health units, located in peri-urban areas of Maputo. The distance from the National Tuberculosis Reference Laboratory in Maputo to Chamanculo General Hospital is 4.5 km and to Polana Caniço General Hospital is 2.3 km. At the time of the evaluation, both laboratories were only performing tuberculosis diagnosis by smear microscopy. Requests for mycobacterial cultures from the two health units were sent to the National Tuberculosis Reference Laboratory in Maputo.

### Patients and specimens

This cross-sectional study was conducted between May 2014 and December 2014. A total of 122 samples, one per patient, categorised as new (i.e. patients with presumptive pulmonary tuberculosis who had never been treated for tuberculosis or had been treated for less than 30 days) or previously-treated (i.e. patients with presumptive pulmonary tuberculosis who had been treated for tuberculosis for more than 30 days), were consecutively included in the study. The specimens were first tested in the health units using the OK method and the remaining samples were shipped to the National Tuberculosis Reference Laboratory in Maputo for mycobacterial culture using the N-acetyl-L-cysteine (NALC)-sodium hydroxide (NaOH)-citrate decontamination method, followed by inoculation in LJ solid media, which was used as a reference standard. Auramine-O staining was also performed at Chamanculo General Hospital, and a Ziehl-Nielsen smear microscopy was performed at Polana Caniço General Hospital.

A 5-day training schedule was performed for laboratory technicians from the two health units, which also included biosafety and OK technical training. Staff technical competency was also evaluated. Standard operational procedures and registration forms were implemented for the study and supervision visits were performed once a week at each participating site. Basic patient demographics were collected from laboratory request forms.

### Ogawa-Kudoh method

The OK method was performed as described by Kudoh and Kudoh.^5^ Briefly, sputum samples were impregnated in a swab. The impregnated swab was placed in a sterile tube containing 3 mL of 4% NaOH solution for 2 minutes and then inoculated with rotary movements in the OK media. Mycobacterial cultures were incubated at 36 ± 1 °C for up to 60 days.

### NALC-NaOH-Citrate/LJ method

Equal volumes of sputum sample and NALC-NaOH-citrate reagent were added to a 50 mL conical tube, after which the mixture was stirred and allowed to stand for 15 min at room temperature. After 15 min, 35 mL 0.067M phosphate buffer (pH 6.8) was added to the mixture. The mixture was then mixed by inversion and centrifuged at 3000 g for 15 min. The supernatant was discarded and the pelleted material was resuspended in 1 mL of buffer. From the suspension, 0.5 mL was inoculated into solid LJ medium and incubated at 37 °C for up to 8 weeks. Tubes were read weekly to verify the presence of mycobacteria.^6^

The use of LJ as the reference standard method, instead of the liquid mycobacterial growth indicator tube culture, which had higher sensitivity and higher contamination rates when compared to LJ, was made in order to establish a direct comparison between the two solid media methods (OK and LJ). A smear microscopy examination of the suspension was also performed.

### Statistical analyses

The culture contribution to diagnostics was calculated based on the recovery rate for both methods, OK and LJ, as the ratio between positive cultures and negative smear microscopy. A database was created in Microsoft Office Access (2007, Version 12; Microsoft Corp, Redmond, Washington, United States), and was later exported to IBM SPSS Statistics for Windows (2015, version 23.0; IBM Corp., Armonk, New York, United States) for analysis of sensitivity, specificity, predictive values (positive and negative) and the degree of agreement between the methods. Concordance between the tests was analysed using the Friedman Kappa test. To verify whether there were associations between the performance of the methods, and the demographic characteristics and categories of patients, the Chi-square test was performed.

The level of statistical significance was set to 0.05 (two-sided) for all analyses. Formulas to calculate sensitivity, specificity, positive predictive value, and negative predictive value were used as previously described.^7^ Levels of agreement were interpreted as follows: values greater than or equal to 0.75 were interpreted as having ‘excellent’ concordance between the two variables; values between 0.4 and 0.75, ‘sufficient to good’ agreement; and values smaller than 0.40, ‘weak’ agreement.

## Results

Of the 122 samples analysed, 60 were from female patients and 62 were from male patients. The median age was 36 years (range:8–70 years). Regarding the category of the patients, most cases had previously been treated (*n* = 84, 68.9%) and 38 cases were new (31.1%) (data not shown in tables).

A total of 98 (80.3%) patients had concordant culture results for both methods, 9 (7.4%) had discordant results and 15 (12.3%) had contaminated results (10 samples were contaminated on LJ only, 4 contaminated on OK only, and 1 was contaminated on both methods). The contamination rate for OK was 4.1% (5/122) and for NALC/LJ, 9.0% (11/122).

Against the NALC/LJ method as the reference standard, and excluding contaminated results, the sensitivity of the OK method was 80% (12/15; 95% CI: 51.4–94.7), the specificity was 93.5% (86/92; 95% CI: 85.8–97.3), the proportion of patients with true-negative results in both techniques was 96.6% (86/89; 95% CI: 89.9–99.1), and the proportion of patients with true-positive results using both techniques was 66.7% (12/18; 95% CI: 41.2–85.6). The agreement between the two methods was Kappa = 0.68 (95% CI: 0.48–0.89) (Table 1).

**TABLE 1:** Comparison between Ogawa-Kudoh and NALC-NaOH/Lowenstein Jensen methods, Mozambique, 2014.

OK Method	NALC-NaOH/LJ					PPV		NPV		Sensitivity		Specificity		Kappa index (concordance)	
	Positive		Negative		Total
	*n*	%	*n*	%		%	95% CI	%	95% CI	%	95% CI	%	95% CI	*n*	95% CI
Positive	12	11.2	6	5.6	18	66.7	41.2–85.6	96.6	89.8–99.1	80	51.4–94.7	93.5	85.8–97.3	0.68	0.48–0.89
Negative	3	2.8	86	80.4	89										
**Total**	**15**	**-**	**92**	**-**	**107†**

OK, Ogawa-Kudoh; LJ, Lowenstein Jensen; PPV, Positive Predictive Value; NPV, Negative Predictive Value.

† , Excluding contamination in both methods; *n* = 15.

For the comparison between the two culture methods, contaminated results were also excluded from the analysis. Mycobacterial culture positivity was 17/111 (15.3%) for NALC/LJ and 18/117 (15.4%) for the OK method. The recovery rate was similar for both methods, 17.1% (18/105) for OK and 17.9% (17/95) for LJ (Tables 2 and 3).

**TABLE 2:** Results ratio of mycobacterial culture and direct microscopy, Mozambique, 2014.†

Ogawa Kudoh method, Health Unit	Ogawa Kudoh result					
	Negative		Positive		Total	
	*n*	%	*n*	%	*n*	%
**Sputum smear**						
Positive	95	81.2	10	8.5	105	89.7
Negative	4	3.4	8	6.8	12	10.3
**Total**	**99**	**84.6**	**18**	**15.4**	**117**	**100.0**

† , Excluding contamination in both methods, *n* = 15.

**TABLE 3:** Results ratio of mycobacterial culture and direct microscopy, Mozambique, 2014.†

NALC/LJ method, National Tuberculosis Reference Laboratory	NALC/LJ result					
	Negative		Positive		Total	
	*n*	%	*n*	%	*n*	%
**Sputum smear**						
Positive	93	83.8	2	1.8	95	85.6
Negative	1	0.9	15	13.5	16	14.4
**Total**	**94**	**84.7**	**17**	**15.3**	**111**	**100.0**

LJ, Lowenstein Jensen.

† , Excluding contamination in both methods, *n* = 15.

## Discussion

In this study, sensitivity, specificity, positive predictive value and negative predictive value were acceptable for the OK method. In addition, there was a high degree of agreement and the recovery rate was similar between the OK and NALC/LJ methods. The contamination rate of the NALC/LJ method was twice as high as the OK method. This finding reinforces the capacity of OK to kill other contaminants, since the OK media is slightly more acidic compared to LJ and neutralises the high concentration of NaOH used to decontaminate the sample. Similar studies performed in Brazil (in 1999, 2006, and 2018) and Peru (in 2007) also found significant differences between the contamination rates obtained with OK and with standard mycobacterial culture methods, again showing that the OK method is efficient in recovering mycobacteria and efficiently killing contaminants.^8,9,10,11^ These results indicate the value of the OK mycobacterial method for diagnosis of pulmonary tuberculosis as a possible alternative for the NALC/LJ method.

The OK mycobacterial method has been used successfully in other countries, particularly in Latin America, including Brazil, as an alternative to traditional culture methods.^4,12,13^ The Pan-American Health Organization recommends the use of OK for tuberculosis diagnosis in areas with limited laboratory capacity.^14^ A study conducted in Uruguay, with the purpose of assessing the efficacy of the OK method for mycobacterial culture in sputum samples, found that after conserving and shipping specimens at room temperature without the addition of any substances to prevent the overgrowth of contaminating bacteria, the OK method was appropriate for culturing mycobacteria, even when processing was delayed for 2–4 days from collection.^15^ Furthermore, the study showed that the duration of the decontamination time was not critical and satisfactory outcomes can still be obtained by increasing the decontamination time up to 4 min.^15^

In general, mycobacterial culture on LJ, when performed on sputum samples, generates approximately 20% more positives than smear microscopy,^4^ because the sensitivity of smears is lower when compared to culture. Access to mycobacterial culture is a challenge in Mozambique, since there are only three tuberculosis reference laboratories, some health units are hard to access by road, the distances are long and the laboratory capacity is limited. Additionally, Mozambique is a tropical country, where the temperature can reach 40 °C during summer. The sample transport system is very mixed and relies on motorbikes, bicycles, ambulances or partners, and samples are not shipped daily. All of these factors contribute to delays in samples reaching the reference laboratories and to sample contamination, leading to inconclusive results.

Although advanced molecular methods, such as the Xpert® MTB/RIF, are available in Mozambique, mycobacterial culture remains the reference standard for tuberculosis diagnosis. Furthermore, culture is needed to isolate the bacteria for other tests, including drug susceptibility tests and sequencing in this environment of increasing resistance to tuberculosis drugs. The mycobacterial culture using the OK method is a simple-to-perform, low-cost method and its biosafety requirements present a lower risk to laboratory technicians, since it does not require agitation or centrifugation steps, and thus reduces the production of aerosols. However, establishment of this assay in peripheral laboratories requires biosafety training and awareness of the risks and precautions for manipulation of Class III pathogens. Additionally, triple packing biosafety procedures must be implemented to ship positive samples from these laboratories to reference laboratories for further diagnostic assays, such as identification of the *M. tuberculosis* complex and drug susceptibility tests.

### Limitations

The present study was conducted at only two health facilities in Maputo City, which might not provide the study with the power to generalise the findings. In addition, there may have been delays in shipping samples from the Health Centres to the National Tuberculosis Reference Laboratory in Maputo, as a result of transportation constraints. This can affect the quality of the specimen, allowing the growth of other bacteria and leading to contamination. Liquid (mycobacterial growth indicator tube) culture results were not evaluated, and were not compared with the OK or LJ mycobacterial culture methods. Liquid culture is known to have higher sensitivity, and could have had implications for the OK or LJ findings. Re-decontamination and re-inoculation of the contaminated tubes, to increase valid results and to reduce contamination rates, was not considered for the present study.

### Conclusion

The OK method showed satisfactory sensitivity and specificity, with lower contamination rates and higher detection rates when compared to the NALC/LJ method. The OK method can effectively be implemented in settings with limited laboratory capacity to isolate tuberculosis bacteria by culture for further testing.
